# Contributions of selenoproteins to breast cancer etiology and racial disparity

**DOI:** 10.1007/s10552-025-02123-y

**Published:** 2026-01-21

**Authors:** Soumen Bera, Li Liu, Weiwei Ma, Ziqiao Xu, Maria Sverdlov, Ryan Deaton, Virgilia Macias, Klara Valyi-Nagy, Andre Kajdacsy-Balla, Kent Hoskins, Elizabeth L. Wiley, Irida Kastrati, Alan M. Diamond

**Affiliations:** 1 Department of Pathology, College of Medicine, Chicago, IL 60612 USA; 2 School of Life Sciences, B.S. Abdur Rahman Crescent Institute of Science and Technology, Chennai, Tamil Nadu India; 3 Department of Epidemiology and Biostatistics, School of Public Health, University of Illinois at Chicago, Chicago, IL 60612 USA; 4 Research Resources Center, University of Illinois at Chicago, Chicago, IL 60612 USA; 5 Division of Hematology and Oncology, University of Illinois at Chicago, Chicago, IL 60612 USA; 6 Department of Cancer Biology, Loyola University Chicago, Maywood, IL 60153 USA

**Keywords:** Selenoproteins, Breast cancer, Polymorphisms, Tissue microarray, Racial disparity

## Abstract

**Purpose:**

Breast cancer etiology is multifactorial with African American women experiencing a significant health disparity in clinical presentation and outcomes. The selenium-containing protein SELENOF has been implicated in breast carcinogenesis by cell culture and animal studies. SELENOF translation is highly regulated in part by the RNA helicase eIF4a3, which binds to the key regulatory regions in the SELENOF mRNA and suppress its translation. In addition, SELENOP, the primary selenium transporter, plays a critical role in selenium delivery to tissues and may influence selenoprotein synthesis. This study aimed to examine the levels of SELENOF and eIF4a3, along with SELENOF and SELENOP genotypes, in breast cancer tissues from African American and Caucasian women

**Methods:**

To study their roles in breast cancer outcome and racial disparity, human tissues were assessed by multiplex immunofluorescence staining with antibodies directed against SELENOF and eIF4a3 and DNA from these tissues were genotyped for previously studied variations in *SELENOF* and the selenium transporter protein *SELENOP*

**Results:**

Elevated levels of both SELENOF and eIF4a3 were observed in breast cancer tissues. SELENOF expression and genotype varied by HER2 status, while *SELENOP* genotypes were associated with breast cancer and showed age-related differences. SELENOF and eIF4a3 were also higher in tissues derived from African American women, who also exhibited higher frequency of a *SELENOP* polymorphism in the non-coding region of the gene

**Conclusion:**

These findings suggest that SELENOF, eIF4a3, and SELENOP may contribute to breast cancer progression and racial disparities in outcomes. Their differential expression and genetic variation highlight potential molecular mechanisms underlying these disparities and may inform future therapeutic or diagnostic strategies.

**Supplementary Information:**

The online version contains supplementary material available at 10.1007/s10552-025-02123-y.

## Introduction

Female breast cancer mortality in the United States has declined, largely due to advancements in early detection and treatment. However, these improvements have not been equitably distributed across racial and ethnic groups, resulting in persistent disparities in outcomes [1, 2]. African American women, in particular, face significantly higher mortality rates and shorter survival times compared to their Caucasian counterparts [3–6]. The multifactorial nature of these disparities presents a complex challenge [7, 8], especially in regions like Chicago, where the gap is more pronounced than the national average [9]. Emerging evidence suggests that biological factors may contribute to these racial disparities, though they remain incompletely understood.

Breast cancer is commonly categorized based on the expression of three molecular markers: estrogen receptor (ER), progesterone receptor (PR), and human epidermal growth factor receptor 2 (Her2). These markers inform both prognosis and treatment strategies. ER/PR-positive tumors, the most prevalent subtype, are typically managed with endocrine therapy, while Her2-positive cancers are treated with targeted anti-Her2 agents. Triple negative breast cancers (ER−, PR−, Her2−) lack targeted therapies and are associated with poorer outcomes. Notably, African American women exhibit a disproportionately high incidence of triple negative breast cancer, estimated at approximately 23% [10], which contributes to their elevated mortality rates. Nevertheless, ER-positive breast cancer remains the most common subtype among African American women. In Chicago, African American patients with ER-positive disease have been reported to have a fourfold higher risk of mortality compared to Caucasians [11]. These findings underscore the possibility of race-related biological differences that transcend tumor subtypes and may inform the development of novel therapeutic approaches to address disparities in breast cancer outcomes.

Selenium has been investigated for its protective role in various cancers, including breast cancer, supported by epidemiological data and animal studies demonstrating its ability to suppress mammary carcinogenesis [12, 13]. Clinical and population-based studies have further linked selenium status to breast cancer survival [14]. Selenium exerts its biological effects primarily through its incorporation into selenoproteins as selenocysteine—the 21st amino acid—encoded by in-frame UGA codons in the mRNAs of 25 known human selenoproteins [15–17]. Among these, SELENOF (formerly SEP15 [18, 19]) has emerged as a candidate implicated in breast cancer, based on initial findings of loss of heterozygosity at the SELENOF locus in breast tumor samples [20]. Unlike housekeeping selenoproteins, SELENOF expression is sensitive to dietary selenium intake and availability [21], suggesting that reduced SELENOF levels due to selenium deficiency may have clinical relevance. Multiple lines of evidence support SELENOF’s involvement in breast cancer etiology, including findings that (1) SELENOF mRNA levels are significantly reduced in late-stage (III and IV) tumors compared to normal breast tissue, (2) diminished SELENOF expression correlates with poor patient prognosis, (3) genetic manipulation of SELENOF in breast cancer cells and murine mammary glands affects cell proliferation and apoptosis, (4) restoration of SELENOF expression mitigates aggressive cancer phenotypes such as clonogenic survival and mammosphere formation, and enhances sensitivity to therapeutic agents, and (5) SELENOF overexpression suppresses tumor growth in murine xenograft models [14, 22, 23]. In this study, we explore the expression levels of SELENOF and eIF4a3, along with genotypes of SELENOF and the selenium transporter SELENOP, in breast cancer tissues from African American and Caucasian women using a tissue microarray (TMA) with relevant clinical data. Clinical outcomes evaluated included tumor grade, stage at presentation, receptor status (ER, PR, HER2), and the proliferation marker Ki67.

## Materials and methods

### Tissue samples

A breast TMA was generated consisting of 141 cases with diverse races, stage and breast cancer subtypes collected at the University of Illinois at Chicago (UIC). Tumors from African American patients (78, 55.3%) are heavily represented in this TMA, reflecting the community served by UIC. There are minimally 5 years of follow-up data and treatment information for all cases. All cases have matched DNA samples; most cases have matched normal tissues (*n* = 60). Additional annotation data include age at surgery (20 to 81-years old), TNM stage, tumor grade, histological type, and clinical IHC/FISH results (ER, PR, HER2, Ki67, p53). Patient demographics are presented in Supplemental Table 1.

### Histology and imaging

The UIC Research Histology Core performed TMA sectioning, assay optimization, and the final staining. The TMA blocks were sectioned at 5 μm, and sections were deparaffinized and stained on BOND RX autostainer (Leica Microsystems). TMA slides were stained with E-cadherin (Opal 690), SELENOF (Opal 570) and eIF4a3 (Opal 520). The slides were scanned at 20× resolution on an Akoya PhenoImager HT using software version 1.0.13 in the whole slide scanning mode with the DAPI, Opal 520, Opal 570, Opal 690 and autofluorescence filter cubes. Images were unmixed using inForm 2.6 and the built-in synthetic libraries. All tissue cores, except those that were significantly fragmented, were included in the analysis. A tissue classifier for Tumor, Non-Tumor and Blank regions was trained, and further analysis was limited to the tumor areas. Nuclei, cytoplasm, and membrane cell compartments were segmented using the adaptive cell segmentation (InForm2.4, Akoya Biosciences) algorithm that considers variations in staining and backgrounds allowing the identification of cellular compartments over multiple image planes. The E-cadherin and SELENOF channels were used to aid cell segmentation. The nuclear, cytoplasmic, membrane and whole cells mean and sum intensities for eIF4a3 and SELENOF as well as membrane E-cadherin were analyzed in areas classified as tumor by the tissue segmentation. Data and markup images were exported for further analysis.

### Genotyping

For single nucleotide polymorphism (SNP) genotyping (see Supplemental Table 2), genomic DNA was isolated from tissue samples and diluted according to the standard protocol and TaqMan genotyping master mix was obtained from ThermoFisher Scientific. Genotyping was conducted using the Roche Light Cycler 96 Real Time PCR instrument, following the manufacturer’s protocol. Briefly, 2 ng of DNA template per reaction was combined with 1X SNP Genotyping Mix and 1X TaqMan Genotyping Master mix in each well of a white 96-well PCR plate (Axygen) in a total volume of 20 µl. PCR was initiated with a pre-incubation step at 95 °C for 10 min, followed by 45 cycles of two-step amplification: denaturation at 95 °C for 10 s and extension at 60 °C for 1 min, with fluorescence signal acquisition for VIC (SELENOF^rs5845A/A^, SELENOP^rs7579C/C^, SELENOP^rs3877899C/C^) and FAM (SELENOF^rs5845G/G^, SELENOP^rs7579T/T^, SELENOP^rs3877899T/T^) reporters. The endpoint genotyping data were acquired and analyzed using Light Cycler 96 software (version 1.1.0.1320) to determine the homozygosity and heterozygosity for the major and minor alleles.

## Statistical analysis

Due to skewness of the SELENOF and eIF4a3 levels, log transformations were done to these variables. In assessing bi-variate relationships (Supplemental Table 1), *p*-values from Pearson correlation coefficients, t-tests or analysis of variance (ANOVA) F-tests, Chi-squared or Fisher’s Exact tests were reported, depending on the types of the variables. Multiple linear regression models were employed to identify factors that were associated with SELENOF and eIF4a3 levels in tumor tissues. Cumulative logistic regression models were used to model ordinal tumor outcomes such as tumor grade and stage. In all regression models, significant factors were first identified using backward model selection method, followed by testing interactions between variables of interest, such as race, SELENOF, eIF4a3, and genotypes. Due to multiple testing for four sets of outcomes, the Bonferroni method to adjust the Type I error probability to be 0.0125 was used. Statistical analyses were performed using SAS (SAS 9.4, SAS Institute, Inc. 2004) and data visualization were conducted in software R (version 4.4.3).

## Results

### Demographics

Information on the histological status of 141 breast cancer patients whose tissues were used as part of this study included patient demographics, receptor status, Ki67 levels, and p53 status were evaluated (Supplemental Table 1) for associations with variables of interests, such as tumor SELENOF levels (in natural log scale, mean = 3.0, SD = 1.7), eIF4a3 levels (in natural log scale, mean = 3.9, SD = 1.2), *SELENOF* genotype (Heterozygote *n* = 56, 39.7%; Homozygote C/G *n* = 51, 36.2%; Homozygote T/A *n* = 34, 24.1%), two *SELENOP* genotypes, as well as tumor grade (Grade 1, Nottingham scores of 3–5, *n* = 31, 22.6%; Grade 2, Nottingham scores 6–7, *n* = 37, 27%; and Grade 3,Nottingham scores of 8–9, *n* = 69, 50.4%) and tumor stage (I *n* = 37, 26.8%; II *n* = 78, 56.5%; III *n* = 22, 15.9%; and IV *n* = 1, 0.7%). The mean age at diagnosis of all patients was 55.8 (SD = 12.9); 78 (55.3%) were non-Hispanic Black, 34 (24.1%) non-Hispanic White. The remaining 29 patients (20.6%) (Hispanic 20, Asian 1, American Indian or Alaska Native 1, and Unknown 7) were categorized as “Other Race” in data analyses. A total of 31 (22.5%) samples were classified as triple negative (ER−, PR−, HER2−) tumors. The percentage of triple negative tumors from non-Hispanic Black women was 21 (26.9%), noticeably higher compared to non-Hispanic White women 4 (11.8%), as expected.

### SELENOF

**Fig. 1:**
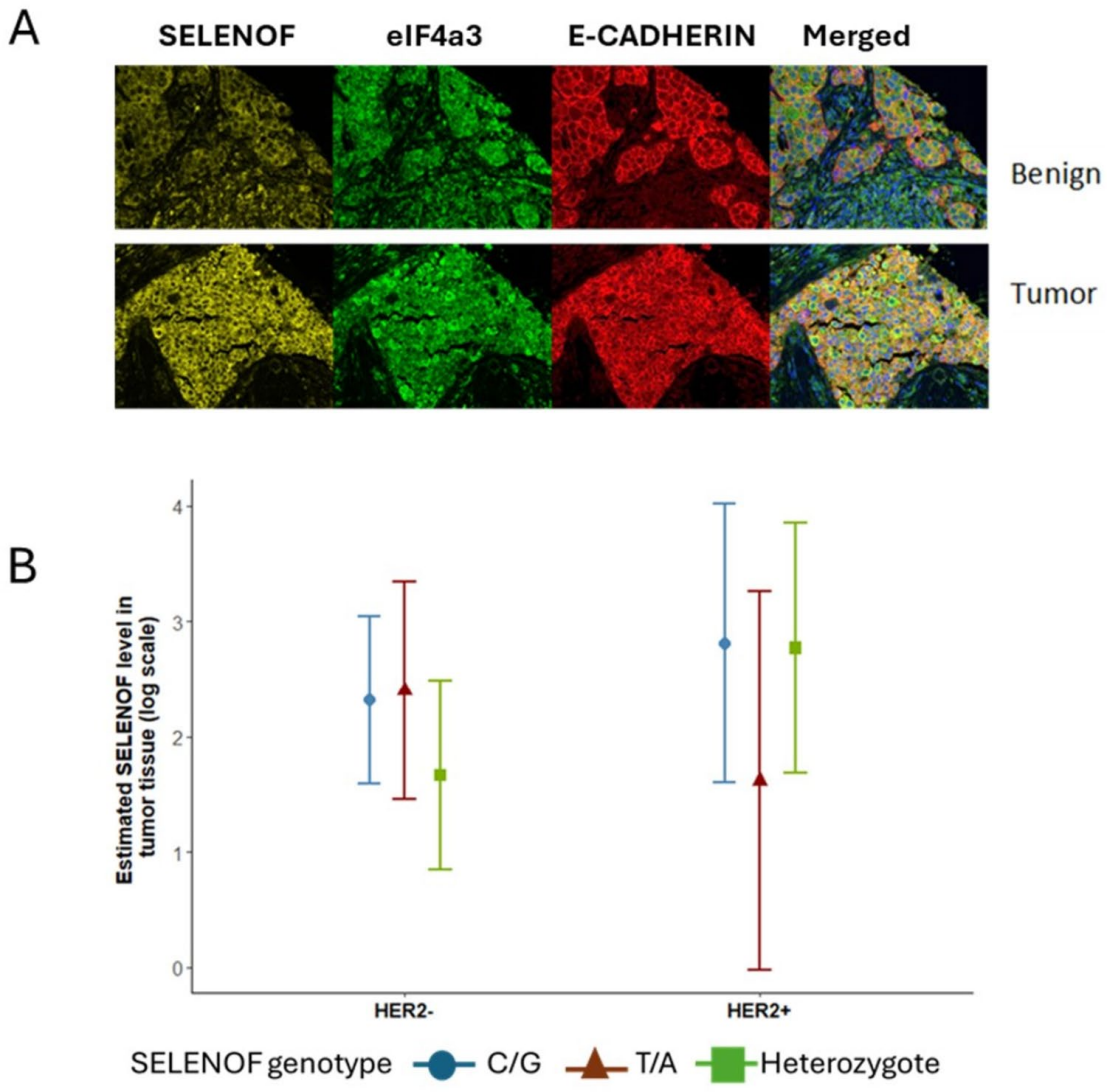
SELENOF and eIF4a3 expression in breast tissue. **A** Representative multiplex immunofluorescence images of SELENOF (yellow), eIF4a3 (green), and E-cadherin (red) in benign (top) and invasive ductal carcinoma (bottom, Grade 1, Nottingham score 3–5) breast tissue from the same African American patient. E-cadherin marks the outer membrane. Images captured at 20X magnification. **B** Interaction effects of HER2 status and SELENOF genotype on SELENOF expression (log-transformed). Blue dots: estimated mean SELENOF level with 99% CI for C/G genotype; red triangles: T/A genotype; green squares: heterozygote genotype. Left panels represent HER2– tumors; right panels represent HER2+ tumors. All estimates are adjusted for covariates in Table 1. (Color figure online)

Our interest in studying SELENOF in breast cancer tissues stems from our previous results indicating that over-expressing SELENOF in breast cancer cells attenuated features of the transformed phenotype and elicited anti-cancer activities, while reducing its levels in cultured cells or mouse mammary glands had the opposite effect, increased proliferation and resistance to cell death [22, 23]. In addition, overexpression of SELENOF in breast cancer cells inhibited *in vivo* tumor growth in a xenograft model [22]. Because SELENOF is heavily regulated at the level of translation, limiting the significance of transcription data on this protein, the relationship between SELENOF and breast cancer was studied in human tissue for which demographics and clinical characteristics were available. Representative images are shown in Fig. 1A. SELENOF staining in breast cancer tissues displayed a pattern consistent with its previously reported localization to the endoplasmic reticulum in other tissue types [24, 25]; however, ER localization was not directly assessed in this study. Samples from both tumor and benign tissues were available from a total of 60 women. Paired t-tests revealed that the SELENOF levels, in natural log scale, were higher in tumor tissue (mean = 2.8, SD = 1.6) as compared to the benign tissue (mean = 2.2, SD = 1.5) from the same individuals (*p*-value < 0.001) and SELENOF levels in tumors were significantly higher in the tumor obtained from African American women (mean = 3.7, SD = 1.4) as compared to other race/ethnicities (non-Hispanic White, mean = 2.3, SD = 1.7; Other race/ethnicity group, mean = 2.2, SD = 1.8, *p*-value < 0.0001, Supplemental Table 1). Multiple regression models for tumor SELENOF levels revealed that, being non-Hispanic Black (*β* = 1.14, *p*-value = 0.0003), having negative PR status (*β* = 0.69, *p*-value = 0.009), lower Ki67 (*β* = −0.52, *p*-value = 0.005), a marker for proliferation, and higher eIF4a3 levels in tumor tissue (β = 0.68, *p* < 0.0001) were associated with higher SELENOF levels (Table 1).

**Table 1:** Multiple linear regression model results for (log) SELENOF levels in tumor tissues

Covariate	Tumor SELENOF level, Natural log scale	
	*β* estimate (SE)	*p*-value
Race		
Non-Hispanic White	Reference	
Non-Hispanic Black	1.14 (0.31)	**0.0003**
Others	−0.25 (0.35)	0.476
PR status		
Positive	Reference	
Negative	0.69 (0.26)	**0.009**
Ki67 level	−0.52 (0.18)	**0.005**
(log) Tumor eIF4a3 level	0.68 (0.09)	**< 0.0001**
SELENOF genotype		
Heterozygote	Reference	
C/G	0.04 (0.54)	0.940
T/A	−1.15 (0.65)	0.081
HER2 status		
Positive	Reference	
Negative	−1.10 (0.40)	**0.006**
SELENOF genotype * HER2		
C/G Negative	0.61 (0.60)	0.315
T/A Negative	1.88 (0.72)	**0.010**

The values in bold indicate statistical significance (p < 0.05)

In addition to quantifying the levels of SELENOF in tissues, the corresponding gene was genotyped to determine the identity of a functional polymorphism in the 3’-untranslated region (3’-UTR) of the SELENOF mRNA [20, 26]. This variant is part of a haplotype that regulates SELENOF translation through an in-frame UGA triplet that serves as the codon for selenocysteine. Moreover, the minor allele has been associated with increased risk of dying from prostate cancer [27] and is approximately 10 times more frequent in the genomes of African Americans as compared to Caucasians [20, 25, 28]. DNA from tissue samples that were included in the TMA were genotyped for the *SELENOF* variation and the minor allele was present much more frequently in the genomes of African American women (29, 37.2%) compared to Caucasians (3, 8.8%, Supplemental Table 1). From the same multivariable regression model for tumor SELENOF levels in Table 1, there were significant interactions between HER2 status and *SELENOF* genotypes, indicating that the relationships between SELENOF levels and SELENOF genotypes differed by HER2 status. Specifically, adjusted for other variables in the model, among HER2-positive patients with T/A genotype, mean SELENOF levels are similarly higher in C/G (estimated difference = 1.19, *p*-value = 0.099) and heterozygote genotypes (estimated difference = 1.15, *p*-value = 0.081). However, this difference did not reach the Bonferroni significance level due to a smaller sample size in our HER2-positive sample (*n* = 31 HER2+, *n* = 107 HER2−). On the contrary, in HER2 negative patients, mean SELENOF level among those expressing the heterozygote genotype were lower than those expressing the C/G genotype (estimated difference = −0.65, *p*-value = 0.030) and T/A (estimated difference = −0.73, *p*-value = 0.027), indicating a statistically significant difference. The differential associations between SELENOF levels and SELENOF genotypes by HER2 status, as revealed by the interactions in Table 1 is presented in Fig. 1B.

### eIF4a3

In order to investigate possible mechanisms of SELENOF regulation in the tissue samples, the TMA was also stained with antibodies to eIF4a3, an RNA helicase primarily involved in RNA splicing [29], but also shown to be elevated by selenium deprivation and negatively regulate SELENOF translation by binding to the SELENOF mRNA 3’-UTR [30]. Paired t-tests revealed higher levels of eIF4a3, in natural log scale, in tumors (mean = 3.8, SD = 1.4) compared to non-tumor tissues (mean = 3.2, SD = 1.3, *p*-value = 0.0004) from the same individual, and eIF4a3 levels were higher in the tumors derived from African Americans (mean = 4.1, SD = 1.1) than Caucasians (mean = 3.4, SD = 1.4, *p*-value = 0.026). Multiple regression models for tumor eIF4a3 levels (Table 2) confirmed the positive association between eIF4a3 and SELENOF.

**Table 2:** Multiple linear regression model results for (log) eIF4a3 levels in tumor tissues

Covariate	Tumor eIF4a3 level, Natural log scale	
	*β* estimate (SE)	*p*-value
(log) Tumor SELENOF level	0.39 (0.05)	**< 0.0001**
ER status		
Positive	Reference	
Negative	0.47 (0.37)	0.200
SELENOP^rs7579^		
Heterozygote	Reference	
Homozygote: C	0.32 (0.24)	0.188
Homozygote: T	0.24 (0.43)	0.578
ER * SELENOP^rs7579^		
Negative Homozygote: C	−1.00 (0.44)	0.024
Negative Homozygote: T	−2.45 (1.15)	0.035

The values in bold indicate statistical significance (p < 0.05)

### SELENOP

Selenoprotein P (SELENOP) contains multiple selenocysteine residues and is a carrier protein that enters the cells of tissues where it is degraded by SecLyase, freeing up selenium for the synthesis of selenoproteins [31, 32]. Since SELENOF translation is regulated by selenium availability at multiple levels, *SELENOP* was genotyped at two polymorphic sites, one within the coding sequence (rs3877899) and another in the regulatory region of the 3’-UTR (rs7579) [33]. It has been speculated that the former regulates the steady state levels of SELENOP while the latter regulates SELENOP levels when selenium is available above the levels consider “adequate”. *SELENOP* polymorphisms in the DNA from various cancer types have also been associated with cancer risk and patient mortality [33, 34].

To assess whether the polymorphisms described above were associated with the clinical and molecular characteristics associated with the patient samples used to generate the TMA, DNA from these samples were genotyped at these loci. The distribution of the SELENOP^rs3877899^ variation, which results in either an alanine or threonine at position 234, was determined, with the heterozygous genotype being associated with a lower stage of breast cancer (Supplemental Table 1, *p-*value = 0.010).

**Fig. 2:**
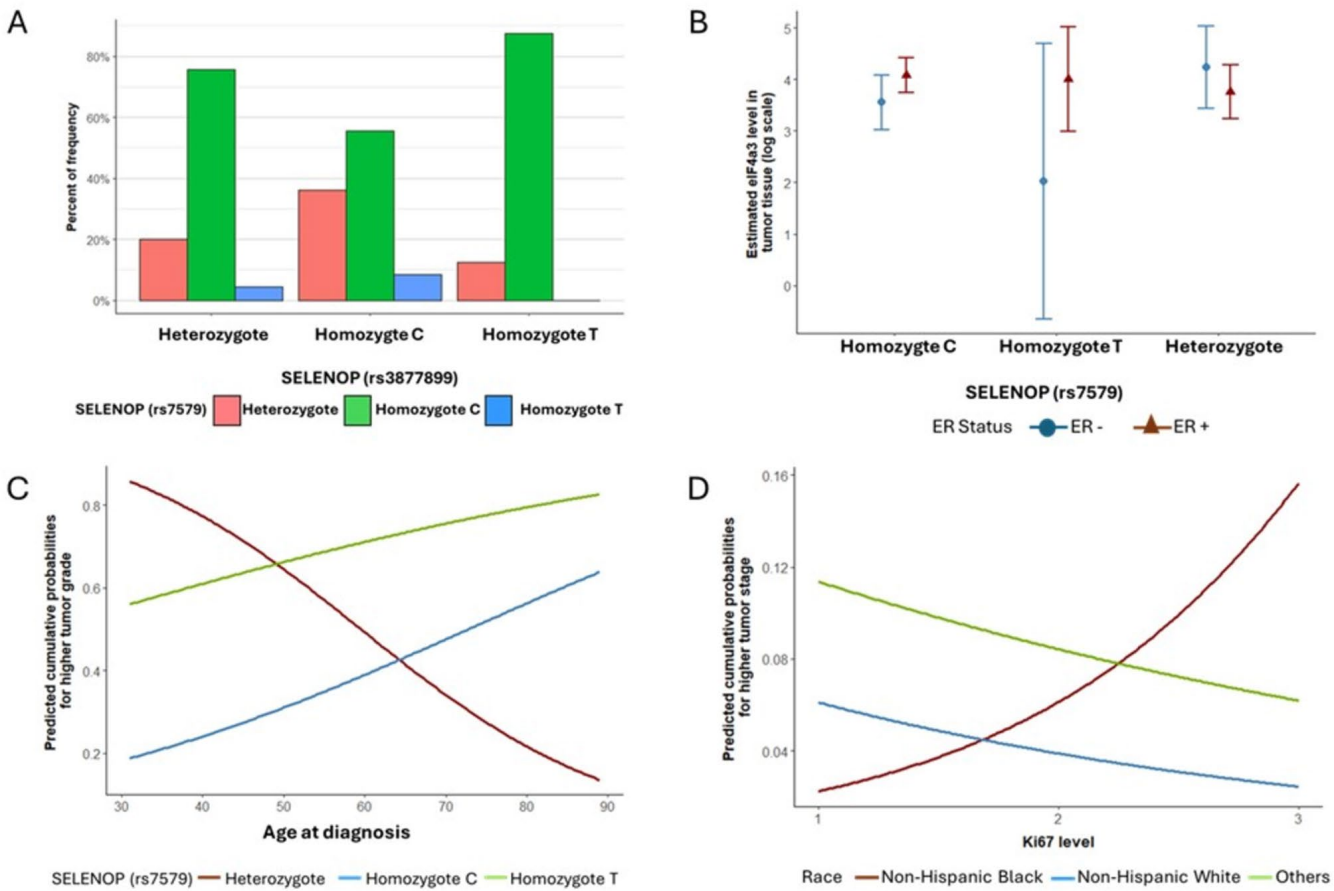
SELENOP genotype associations and tumor characteristics. **A** Distribution of *SELENOP* ^rs7579^ genotypes across *SELENOP *^rs387789^9 genotypes. **B** Interaction effects of ER status and *SELENOP* ^rs7579^ genotype on eIF4a3 expression (log-transformed and estimates shown with 99% CI. **C** Age-by-*SELENOP* ^rs7579^ genotype interaction effects on probability of higher tumor grade (Nottingham score). Estimates adjusted for covariates in Table 3. **D** Ki67-by-race interaction effects on probability of higher tumor stage (Stage III). Estimates adjusted for covariates in Table 4

In contrast to the distribution of the *SELENOP* ^rs3877899^ TT variation, which was represented in the DNA of 17% of the tissue samples, the *SELENOP*^rs7579^ variation TT genotype at position 25,191 located within the 3’-UTR of the SELENOP cDNA was represented in only approximately 5.7% of tissue samples and the GG homozygous allele was more prevalent among the DNAs from samples obtained from African Americans than Caucasian women (78.2% vs. 52.9%, *p*-value = 0.006). There was also a statistically significant association between the identity of the polymorphisms at both *SELENOP* loci (*p*-value = 0.033). Specifically, the *SELENOP*^rs7579^ homozygote C genotype was more prevalent (87.5%) in DNAs expressing the *SELENOP*^rs3877899^ homozygote T genotype than the other two *SELENOP* ^rs3877899^ genotypes; *SELENOP*^rs7579^ heterozygotes were more prevalent (36.1%) in *SELENOP* ^rs3877899^ homozygote C genotypes than the other two SELENOP ^rs3877899^ genotypes (Fig. 2A).

Although mean eIF4a3 levels were not different by either *SELENOP* genotypes (Supplemental Table 1), multiple regression modeling for tumor eIF4a3 levels revealed that expressing the *SELENOP*^rs7579^ genotype significantly modified the association between ER status and tumor eIF4a3 levels (Table 2, 2 degrees of freedom interaction *p*-value = 0.024). The interaction effect between ER status and *SELENOP* ^rs7579^ genotype for eIF4A3 levels is shown in Fig. 2B. While there was a minimal difference in ER status in samples expressing the heterozygote genotype (*p*-value = 0.200), mean eIF4a3 levels were significantly higher in ER+ tumors among those with a homozygote C genotype (estimated difference = 0.52, *p*-value = 0.029), and with a homozygote T genotype (estimated difference = 1.97), although the *p-*value was 0.072 due to the very small sample size for homozygote T carriers (*n* = 8, 5.7%).

## Associations of eIF4a3 and SELENOP on associated tumor outcomes

### Tumor grade

Multivariate ordinal logistic regression models (Table 3) confirmed that triple negative tumors (OR = 5.26, 99% CI = 0.97–28.51, *p*-value** = **0.011) and higher Ki67 levels (OR = 14.76, 99% CI = 4.98–43.79, *p*-value < 0.0001) were associated with higher tumor grades. Each unit increase in natural log scale of eIF4a3 resulted in an OR of 1.59 for higher-grade of tumor (99% CI = 1.02–2.50, *p*-value = 0.008). Age-by-*SELENOP*^rs7579^ analysis indicated a significant interaction of age on tumor grade by *SELENOP*^rs7579^ genotypes (Fig. 2C). Specifically, a higher age showed a borderline protective effect on tumor grade in *SELENOP* heterozygotes (*p* = 0.051), while the association with age was reversed among homozygotes. For women of 40 years, having a heterozygote genotype increased the odds of having a higher-grade cancer to 10.77 times (*p*-value = 0.005), compared to a 40-year-old expressing the homozygote C genotype. In contrast, an 85-year-old woman expressing the homozygote C genotype exhibited an increased chance of having higher tumor grade by 7.62 times compared to someone with a heterozygote genotype (*p*-value = 0.079).

**Table 3:** Cumulative logit logistic regression results for higher tumor grade

Covariate	Higher tumor grade		
	Estimate (SE)	OR (99% CI)	*p*-value
(log) Tumor eIF4a3 level	0.47 (0.17)	1.59 (1.02–2.50)	**0.008**
Ki67 level	2.69 (0.42)	14.76 (4.98–43.79)	**< 0.0001**
Triple negative			
No	Reference		
Yes	1.66 (0.66)	5.26 (0.97–28.51)	**0.011**
SELENOP^rs7579^			
Heterozygote	Reference		
Homozygote: C	−6.30 (2.29)		**0.006**
Homozygote: T	−4.20 (5.08)		0.408
Age	−0.06 (0.03)		0.051
Age * SELENOP^rs7579^			
Homozygote: C	0.10 (0.04)		**0.011**
Homozygote: T	0.09 (0.09)		0.352

The values in bold indicate statistical significance (p < 0.05)

### Tumor stage

Multivariate ordinal logistic regression models (Table 4) revealed that higher eIF4a3 levels are associated with higher tumor stage (OR = 1.64, 99% CI = 1.10–2.46, *p*-value = 0.002). *SELENOP* ^rs3877899^ homozygote C (OR = 3.11, 99% CI = 1.04–9.29, *p*-value = 0.008) and T (OR = 3.25, 99% CI = 0.77–13.72, *p*-value = 0.035) genotypes were both associated with a more advanced cancer stage compared to the heterozygotes. Significant Ki67 and race interactions indicated that the association between Ki67 and tumor stage differed between African Americans and Caucasians (*p*-value = 0.018). Specifically, among African American patients, higher Ki67 was associated with higher tumor stage (OR = 2.84, *p* = 0.005), while this effect was insignificant for both Caucasians and other races (Fig. 2D).

**Table 4:** Cumulative logit logistic regression results for higher tumor stage

Covariate	Higher tumor stage		
	Estimate (SE)	OR (99% CI)	*p*-value
(log) Tumor eIF4a3 level	0.50 (0.16)	1.64 (1.10–2.46)	**0.002**
SELENOP ^rs3877899^			
Heterozygote			
Homozygote: C	1.13 (0.42)	3.11 (1.04–9.29)	**0.008**
Homozygote: T	1.18 (0.56)	3.25 (0.77–13.72)	0.035
Race			
Non-Hispanic White			
Non-Hispanic Black	−2.56 (1.27)		0.044
Others	0.54 (1.76)		0.760
Ki67 level	−0.47 (0.52)		0.360
Ki67 level * Race			
Non-Hispanic Black	1.52 (0.64)		**0.018**
Others	0.14 (0.84)		0.865

The values in bold indicate statistical significance (p < 0.05)

## Discussion

Herein, we investigated relationships among components of the selenoprotein regulatory pathway and the presentation and etiology of breast cancer among African American and Caucasian women. Selenium ingested either in the diet or by the consumption of supplements is converted to SELENOP in the liver, which enters the circulation and is delivered to organs where it is degraded, and the selenium is then used for the synthesis of selenoproteins such as SELENOF. These selenoproteins then perform a host of essential and non-essential functions with several selenoproteins implicated in cancer risk and outcome [16]. This process is highly regulated at the level of translation, with the synthesis of approximately half of the 25 human selenoproteins being influenced by selenium availability and the selenium-dependent modification of the selenocysteyl-tRNA [35, 36] and the response to selenium levels by the selenoprotein translational inhibitor eIF4a3, whose over-expression during selenium deficiency inhibits the translation of some selenoproteins eIF4a3 [30, 37]. Given our previous data indicating that SELENOF levels are reduced in some cancers [25], increasing or decreasing SELENOF can alter the transformation-related phenotype of breast cancer cells and SELENOF over-expression can attenuate the growth of tumors in mouse xenograft studies [14, 22, 23], we examined the relationship of components of the selenium pathway using a human breast cancer TMA.

Given our previous data indicating the significant loss of SELENOF in prostate cancer and the much higher frequency of the minor *SELENOF* allele in the genomes of African Americans predicted to be associated with reduced SELENOF synthesis, observing its elevation in breast cancer was unexpected. SELENOF levels were associated with being negative for the progesterone receptor and higher levels of the proliferation marker Ki67, as well as being elevated in tissue obtained from African American women as compared to those from Caucasians. The expected relationship between the minor *SELENOF* allele and lower SELENOF levels was only observed in HER2-positive tumor tissues, and this relationship was not seen in HER-negative tissues, although he relationship failed to reach statistical significance due to correction for multiple comparisons. The analysis also revealed that eIF4a3 levels were elevated in breast cancer compared to benign tissue and was associated with higher tumor stage at presentation ER+ tumors expressing homozygote *SELENOP* genotypes. The frequent observation that eiF4a3 is elevated in cancer indicates it may serve as an oncogene when over-expressed, but it is unknown which of the protein’s functions, such as participating in RNA metabolism or translational regulation of selenoproteins might contribute to malignancy. Given the established ability of eiF4a3 to attenuate SELENOF translation, it was unexpected that both eIF4a3 and SELENOF levels were elevated in breast cancers. Additional studies are required to discern the multiple mechanisms of SELENOF regulation and what differences in this regulation may occur in distinct tissues and cancers.

The analysis of the tissues included in the TMA also indicated several significant differences between the status of the examined variable between African Americans and Caucasian tissue samples. As previously reported by others [10], the percentage of triple negative breast cancers was higher in African American women. In addition, the association of higher Ki67 and tumor stage was only significant among samples from African Americans. Regarding markers associated with selenoprotein regulation, we found that both SELENOF and eIF4a3 levels were higher in cancer tissues obtained from African Americans. Also of note was the more frequent distribution of the polymorphism in the gene for the transporter of selenium, SELENOP, in the 3’-UTR regulatory region that may contribute to determining the levels of SELENOP in tissues in response to the dietary intake of the trace element [33]. This may be significant as there have been several reports of lower selenium levels in African American populations, including those in Chicago from whose tissues were used to generate the TMA described here [25, 38–40]. Whether any of these observations contribute to the disparity in breast cancer etiology and mortality remains unclear. Conclusion: These findings indicate likely differential roles for SELENOF in cancer biology and demonstrate eIF4a3 as a potential oncogene. The intricate relationship between genetic variations, ER/PR/HER2 status, and selenoprotein regulations may support precision treatment and targeted therapy. SELENOF and eIF4a3 status may also serve as biomarkers or therapeutic targets, but large-scale clinical studies are needed to validate these associations.

## Supplementary Information

Below is the link to the electronic supplementary material.

Supplementary file1 (DOCX 53 KB)

## Data Availability

The datasets generated and analyzed during this study are available from the corresponding author upon reasonable request. These include raw and processed multiplex immunofluorescence data for SELENOF and eIF4a3 expression in breast tissue samples, genotyping data for SELENOF and SELENOP variants, associated clinical metadata (e.g., HER2 status, age, race) used in statistical analyses. More than one author has directly accessed and verified the accuracy and integrity of the data reported in this manuscript, and all authors have reviewed and approved the final version.
