# Supplementary material for: Contributions of selenoproteins to breast cancer etiology and racial disparity
Source: Cancer Causes Control. 2026 Jan 21;37(2):35. doi: 10.1007/s10552-025-02123-y (PMC12823618; doi:10.1007/s10552-025-02123-y)
Supplement: Supplementary file 1 — Supplementary file1 (DOCX 53 KB) [file 10552_2025_2123_MOESM1_ESM.docx]

**Supplemental Table 1. Summary statistics (mean (SD), n (%)) for variables of interests by patient characteristics**

| **Characteristic** | **Total*^1^***  **N=141** | **SELENOF level in tumor tissue (log scale)** | | **eIF4a3 level in tumor tissue (log scale)** | | **SELENOF genotype*^1^*** | | | | **SELENOP^rs3877899^ *^1^*** | | | | **SELENOP^rs7579^ *^1^*** | | | |
| --- | --- | --- | --- | --- | --- | --- | --- | --- | --- | --- | --- | --- | --- | --- | --- | --- | --- |
|  |  | **Mean (SD)** | ***p*-value** | **Mean (SD)** | ***p*-value** | **Heterozygote**  **(n=56, 39.7%)** | **C/G**  **(n=51, 36.2%)** | **T/A**  **(n=34, 24.1%)** | ***p*-value** | **Heterozygote**  **(n=45, 31.9%)** | **Homozygote: C**  **(n=72, 51.1%)** | **Homozygote: T**  **(n=24, 17.0%)** | ***p*-value** | **Heterozygote**  **(n=38, 27.0%)** | **Homozygote: C**  **(n=95, 67.4%)** | **Homozygote: T**  **(n=8, 5.7%)** | ***p*-value** |
| **Age at diagnosis, mean (SD)** | 55.8 (12.9) | -0.03*^2^* | 0.708 | 0.02*^2^* | 0.858 | 54.5 (12.2) | 56.5 (12.6) | 56.8 (14.4) | 0.651*^4^* | 55.4 (12.9) | 56.3 (12.6) | 55.2 (14.1) | 0.902*^4^* | 56.7 (14.0) | 55.5 (12.7) | 55.1 (9.8) | 0.882*^4^* |
|  |  |  |  |  |  |  |  |  |  |  |  |  |  |  |  |  |  |
| **Race and ethnicity** |  |  | **<0.0001***^4^* |  | 0.026*^4^* |  |  |  | **0.0003***^6^* |  |  |  | 0.075*^5^* |  |  |  | **0.006***^6^* |
| Non-Hispanic Black | 78 (55.3) | 3.7 (1.4) |  | 4.1 (1.1) |  | 31 (39.7) | 18 (23.1) | 29 (37.2) |  | 30 (38.5) | 38 (48.7) | 10 (12.8) |  | 16 (20.5) | 61 (78.2) | 1 (1.3) |  |
| Non-Hispanic White | 34 (24.1) | 2.3 (1.7) |  | 3.4 (1.4) |  | 12 (35.3) | 19 (55.9) | 3 (8.8) |  | 9 (26.5) | 20 (58.8) | 5 (14.7) |  | 12 (35.3) | 18 (52.9) | 4 (11.8) |  |
| Others | 29 (20.6) | 2.2 (1.8) |  | 3.9 (1.3) |  | 12 (44.4) | 13 (48.1) | 2 (7.4) |  | 5 (18.5) | 13 (48.1) | 9 (33.3) |  | 10 (37.0) | 14 (51.9) | 3 (11.1) |  |
|  |  |  |  |  |  |  |  |  |  |  |  |  |  |  |  |  |  |
| **ER status** |  |  | 0.619*^3^* |  | 0.371*^3^* |  |  |  | 0.658*^5^* |  |  |  | 0.833*^5^* |  |  |  | 0.662*^6^* |
| Negative | 41 (29.7) | 3.2 (1.8) |  | 3.8 (1.2) |  | 16 (39.0) | 13 (31.7) | 12 (29.3) |  | 13 (31.7) | 20 (48.8) | 8 (19.5) |  | 11 (26.8) | 29 (70.7) | 1 (2.4) |  |
| Positive | 97 (70.3) | 3.0 (1.7) |  | 4.0 (1.2) |  | 38 (39.2) | 37 (38.1) | 22 (22.7) |  | 31 (32.0) | 51 (52.6) | 15 (15.5) |  | 27 (27.8) | 63 (64.9) | 7 (7.2) |  |
|  |  |  |  |  |  |  |  |  |  |  |  |  |  |  |  |  |  |
| **PR status** |  |  | 0.245*^3^* |  | 0.343*^3^* |  |  |  | 0.662*^5^* |  |  |  | 0.682*^5^* |  |  |  | 0.405*^6^* |
| Negative | 49 (35.5) | 3.3 (1.7) |  | 3.8 (1.4) |  | 17 (34.7) | 20 (40.8) | 12 (24.5) |  | 15 (30.6) | 24 (49.0) | 10 (20.4) |  | 15 (30.6) | 33 (67.3) | 1 (2.0) |  |
| Positive | 89 (64.5) | 2.9 (1.7) |  | 4.0 (1.2) |  | 37 (41.6) | 30 (33.7) | 22 (24.7) |  | 29 (32.6) | 47 (52.8) | 13 (14.6) |  | 23 (25.8) | 59 (66.3) | 7 (7.9) |  |
|  |  |  |  |  |  |  |  |  |  |  |  |  |  |  |  |  |  |
| **HER2 status** |  |  | 0.282*^3^* |  | 0.622*^3^* |  |  |  | 0.230*^5^* |  |  |  | 0.886*^5^* |  |  |  | 0.568*^6^* |
| Negative | 107 (77.5) | 3.0 (1.7) |  | 3.9 (1.2) |  | 38 (35.5) | 40 (37.4) | 29 (27.1) |  | 33 (30.8) | 56 (52.3) | 18 (16.8) |  | 27 (25.2) | 73 (68.2) | 7 (6.5) |  |
| Positive | 31 (22.5) | 3.3 (1.6) |  | 4.0 (1.3) |  | 16 (51.6) | 10 (32.3) | 5 (16.1) |  | 11 (35.5) | 15 (48.4) | 5 (16.1) |  | 11 (35.5) | 19 (61.3) | 1 (3.2) |  |
|  |  |  |  |  |  |  |  |  |  |  |  |  |  |  |  |  |  |
| **Triple negative** |  |  | 0.647*^3^* |  | 0.184*^3^* |  |  |  | 0.469*^5^* |  |  |  | 0.602*^5^* |  |  |  | 0.642*^6^* |
| No | 107 (77.5) | 3.1 (1.7) |  | 4.0 (1.2) |  | 42 (39.3) | 41 (38.3) | 24 (22.4) |  | 35 (32.7) | 56 (52.3) | 16 (15.0) |  | 31 (29.0) | 69 (64.5) | 7 (6.5) |  |
| Yes | 31 (22.5) | 2.9 (1.9) |  | 3.6 (1.3) |  | 12 (38.7) | 9 (29.0) | 10 (32.3) |  | 9 (29.0) | 15 (48.4) | 7 (22.6) |  | 7 (22.6) | 23 (74.2) | 1 (3.2) |  |
|  |  |  |  |  |  |  |  |  |  |  |  |  |  |  |  |  |  |
| **Ki67 Status** |  |  | 0.820*^4^* |  | 0.834*^4^* |  |  |  | 0.314*^5^* |  |  |  | 0.865*^5^* |  |  |  | 0.480*^6^* |
| 0-10% | 29 (21.5) | 3.2 (1.5) |  | 4.0 (1.3) |  | 13 (44.8) | 10 (34.5) | 6 (20.7) |  | 8 (27.6) | 16 (55.2) | 5 (17.2) |  | 10 (34.5) | 16 (55.2) | 3 (10.3) |  |
| 11-50% | 69 (51.1) | 3.0 (1.8) |  | 3.9 (1.2) |  | 22 (31.9) | 30 (43.5) | 17 (24.6) |  | 25 (36.2) | 33 (47.8) | 11 (15.9) |  | 16 (23.2) | 49 (71.0) | 4 (5.8) |  |
| >50% | 37 (27.4) | 3.0 (1.8) |  | 4.0 (1.2) |  | 18 (48.6) | 9 (24.3) | 10 (27.0) |  | 10 (27.0) | 20 (54.1) | 7 (18.9) |  | 11 (29.7) | 25 (67.6) | 1 (2.7) |  |
|  |  |  |  |  |  |  |  |  |  |  |  |  |  |  |  |  |  |
| **p53 Status** |  |  | 0.998*^3^* |  | 0.974*^3^* |  |  |  | 0.797*^5^* |  |  |  | 0.304*^6^* |  |  |  | 0.415*^6^* |
| Not over expressed (<20 staining) | 47 (56.0) | 2.9 (1.8) |  | 3.8 (1.2) |  | 17 (36.2) | 18 (38.3) | 12 (25.5) |  | 18 (38.3) | 26 (55.3) | 3 (6.4) |  | 15 (31.9) | 28 (59.6) | 4 (8.5) |  |
| Over Expressed (>20 staining) | 37 (44.0) | 2.9 (1.4) |  | 3.8 (1.2) |  | 16 (43.2) | 13 (35.1) | 8 (21.6) |  | 9 (24.3) | 23 (62.2) | 5 (13.5) |  | 7 (18.9) | 27 (73.0) | 3 (8.1) |  |
|  |  |  |  |  |  |  |  |  |  |  |  |  |  |  |  |  |  |
| **Tumor stage** |  |  | 0.151*^4^* |  | **0.007***^4^* |  |  |  | 0.389*^5^* |  |  |  | **0.010***^6^* |  |  |  | 0.555*^6^* |
| I | 37 (26.8) | 2.6 (1.8) |  | 3.4 (1.4) |  | 17 (45.9) | 9 (24.3) | 11 (29.7) |  | 20 (54.1) | 15 (40.5) | 2 (5.4) |  | 12 (32.4) | 23 (62.2) | 2 (5.4) |  |
| II | 78 (56.5) | 3.1 (1.6) |  | 4.0 (1.2) |  | 30 (38.5) | 32 (41.0) | 16 (20.5) |  | 19 (24.4) | 41 (52.6) | 18 (23.1) |  | 18 (23.1) | 54 (69.2) | 6 (7.7) |  |
| III/IV^#^ | 23 (16.7) | 3.4 (1.7) |  | 4.3 (1.0) |  | 7 (30.4) | 9 (39.1) | 7 (30.4) |  | 5 (21.7) | 14 (60.9) | 4 (17.4) |  | 8 (34.8) | 15 (65.2) | 0 |  |
|  |  |  |  |  |  |  |  |  |  |  |  |  |  |  |  |  |  |
| **Tumor grade** |  |  | 0.507*^4^* |  | 0.501*^4^* |  |  |  | 0.882*^5^* |  |  |  | 0.633*^6^* |  |  |  | 0.978*^6^* |
| Grade 1- Nottingham score 3-5 | 31 (22.6) | 2.9 (1.4) |  | 3.7 (1.3) |  | 14 (45.2) | 11 (35.5) | 6 (19.4) |  | 11 (35.5) | 16 (51.6) | 4 (12.9) |  | 7 (22.6) | 22 (71.0) | 2 (6.5) |  |
| Grade 2- Nottingham score 6-7 | 37 (27.0) | 2.8 (1.9) |  | 3.8 (1.2) |  | 13 (35.1) | 13 (35.1) | 11 (29.7) |  | 13 (35.1) | 20 (54.1) | 4 (10.8) |  | 11 (29.7) | 24 (64.9) | 2 (5.4) |  |
| Grade 3- Nottingham score 8-9 | 69 (50.4) | 3.2 (1.7) |  | 4.0 (1.2) |  | 27 (39.1) | 25 (36.2) | 17 (24.6) |  | 19 (27.5) | 35 (50.7) | 15 (21.7) |  | 20 (29.0) | 45 (65.2) | 4 (5.8) |  |
|  |  |  |  |  |  |  |  |  |  |  |  |  |  |  |  |  |  |
| **SELENOF level in tumor tissue (log scale), mean (SD)** | 3.0 (1.7) | - | - | 0.55*^2^* | **<0.0001** | 2.6 (1.7) | 3.2 (1.7) | 3.5 (1.6) | 0.063*^4^* | 3.2 (1.7) | 2.8 (1.8) | 3.5 (1.6) | 0.125*^4^* | 2.7 (1.8) | 3.3 (1.7) | 2.1 (1.4) | 0.062*^4^* |
|  |  |  |  |  |  |  |  |  |  |  |  |  |  |  |  |  |  |
| **eIF4a3 level in tumor tissue (log scale), mean (SD)** | 3.9 (1.2) | 0.55*^2^* | **<0.0001** | - | - | 3.6 (1.3) | 4.0 (1.1) | 4.1 (1.3) | 0.158*^4^* | 4.0 (1.2) | 3.8 (1.2) | 4.1 (1.4) | 0.395*^4^* | 3.8 (1.4) | 4.0 (1.2) | 3.4 (1.3) | 0.313*^4^* |
|  |  |  |  |  |  |  |  |  |  |  |  |  |  |  |  |  |  |
| **SELENOF genotype** |  |  | 0.063*^4^* |  | 0.158*^4^* |  |  |  | - |  |  |  | 0.490*^5^* |  |  |  | 0.171*^6^* |
| Heterozygote | 56 (39.7) | 2.6 (1.7) |  | 3.6 (1.3) |  | - | - | - |  | 17 (30.4) | 29 (51.8) | 10 (17.9) |  | 18 (32.1) | 36 (64.3) | 2 (3.6) |  |
| C/G | 51 (36.2) | 3.2 (1.7) |  | 4.0 (1.1) |  | - | - | - |  | 13 (25.5) | 29 (56.9) | 9 (17.6) |  | 12 (23.5) | 33 (64.7) | 6 (11.8) |  |
| T/A | 34 (24.1) | 3.5 (1.6) |  | 4.1 (1.3) |  | - | - | - |  | 15 (44.1) | 14 (41.2) | 5 (14.7) |  | 8 (23.5) | 26 (76.5) | 0 |  |
|  |  |  |  |  |  |  |  |  |  |  |  |  |  |  |  |  |  |
| **SELENOP^rs3877899^** |  |  | 0.125*^4^* |  | 0.395*^4^* |  |  |  | 0.490*^5^* |  |  |  | - |  |  |  | 0.033*^6^* |
| Heterozygote | 45 (31.9) | 3.2 (1.7) |  | 4.0 (1.2) |  | 17 (37.8) | 13 (28.9) | 15 (33.3) |  | - | - | - |  | 9 (20.0) | 34 (75.6) | 2 (4.4) |  |
| Homozygote: C | 72 (51.1) | 2.8 (1.8) |  | 3.8 (1.2) |  | 29 (40.3) | 29 (40.3) | 14 (19.4) |  | - | - | - |  | 26 (36.1) | 40 (55.6) | 6 (8.3) |  |
| Homozygote: T | 24 (17.0) | 3.5 (1.5) |  | 4.1 (1.3) |  | 10 (41.7) | 9 (37.5) | 5 (20.8) |  | - | - | - |  | 3 (12.5) | 21 (87.5) | 0 |  |
|  |  |  |  |  |  |  |  |  |  |  |  |  |  |  |  |  |  |
| **SELENOP^rs7579^** |  |  | 0.062*^4^* |  | 0.313*^4^* |  |  |  | 0.171*^6^* |  |  |  | 0.033*^6^* |  |  |  | - |
| Heterozygote | 38 (27.0) | 2.7 (1.8) |  | 3.8 (1.4) |  | 18 (47.4) | 12 (31.6) | 8 (21.1) |  | 9 (23.7) | 26 (68.4) | 3 (7.9) |  | - | - | - |  |
| Homozygote: C | 95 (67.4) | 3.3 (1.6) |  | 4.0 (1.2) |  | 36 (37.9) | 33 (34.7) | 26 (27.4) |  | 34 (35.8) | 40 (42.1) | 21 (22.1) |  | - | - | - |  |
| Homozygote: T | 8 (5.7) | 2.1 (1.4) |  | 3.4 (1.2) |  | 2 (25.0) | 6 (75.0) | 0 |  | 2 (25.0) | 6 (75.0) | 0 |  | - | - | - |  |

*^1^*n (%)

Statistical methods: *^2^*Pearson Correlation Coefficient; *^3^*Two sample t-test; *^4^*One-way ANOVA F test; *^5^*Chi-Squared test; *^6^*Fisher’s Exact test.

^#^ 22 patients expressing tumor stage III, 1 patient had tumor stage IV.

Continuous variables were expressed as mean (SD) and compared using two sample t-test or one-way ANOVA as appropriate. Categorical variables were presented as count (%) and evaluated by Chi-squared test or Fisher’s exact test for sparse cell counts. The correlation between two continuous variables were measured by Pearson correlation coefficient. *P*-value<0.0125 was considered significant after Bonferroni correction.

**Supplemental Table 2.**

| Gene | SNP ID | Assay ID | Allele |
| --- | --- | --- | --- |
| SELENOF | rs5845 | C____876937_1_ | A/G |
| SEPP1 | rs3877899 | C___2841533_10 | C/T |
|  | rs7579 | C___8806056_10 | C/T |
